# Individual and Community Socioeconomic Status: Impact on Mental Health in Individuals with Arthritis

**DOI:** 10.1155/2014/256498

**Published:** 2014-08-04

**Authors:** Chivon A. Mingo, Kathryn R. Martin, Jack Shreffler, Britta Schoster, Leigh F. Callahan

**Affiliations:** ^1^Gerontology Institute, Georgia State University, P.O. Box 3984, Atlanta, GA 30302, USA; ^2^Epidemiology Group, School of Medicine and Dentistry, University of Aberdeen, Aberdeen AB25 2ZD, UK; ^3^Thurston Arthritis Research Center, University of North Carolina at Chapel Hill, Chapel Hill, NC 27599, USA; ^4^Departments of Medicine and Social Medicine, Thurston Arthritis Research Center, Department of Epidemiology, Cecil G. Sheps Center for Health Services Research, University of North Carolina at Chapel Hill, Chapel Hill, NC 27599, USA

## Abstract

To examine the impact of individual and community socioeconomic status (SES) measures on mental health outcomes in individuals with arthritis, participants with self-reported arthritis completed a telephone survey assessing health status, health attitudes and beliefs, and sociodemographic variables. Regression analyses adjusting for race, gender, BMI, comorbidities, and age were performed to determine the impact of individual and community level SES on mental health outcomes (i.e., Medical Outcomes Study SF-12v2 mental health component, the Centers for Disease Control and Prevention Health-Related Quality of Life Healthy Days Measure, Center for Epidemiological Studies Depression [CES-D] scale). When entered singly, lower education and income, nonmanagerial occupation, non-homeownership, and medium and high community poverty were all significantly associated with poorer mental health outcomes. Income, however, was more strongly associated with the outcomes in comparison to the other SES variables. In a model including all SES measures simultaneously, income was significantly associated with each outcome variable. Lower levels of individual and community SES showed most consistent statistical significance in association with CES-D scores. Results suggest that both individual and community level SES are associated with mental health status in people with arthritis. It is imperative to consider how interventions focused on multilevel SES factors may influence existing disparities.

## 1. Introduction

Arthritis, the leading cause of disability in the United States, often results in pain and functional limitations [1]. Arthritis is also associated with negative psychological responses such as an increase in anxiety, depression, lower health-related quality of life (HRQOL), and feelings of helplessness [2, 3]. In fact, studies have reported that the odds of having a mental disorder are significantly higher for people with arthritis than without, particularly among diagnoses of mood and anxiety disorders [4]. Because the negative psychological impact of arthritis can be high, it is imperative to identify and understand factors that may contribute to disease burden and poor health-related quality of life.

Moreover, individuals with low SES are more likely to be depressed or have poor mental health symptoms [5, 6]. Yet, the majority of studies examining the relationship between SES and health outcomes, including arthritis studies, have focused mainly on physical health outcomes [7–9]. In fact, the literature is replete with research examining the relationship between various measures of socioeconomic status (SES) and chronic disease outcomes [10]. The relationship between lower levels of SES, both at the individual and community level, and poorer health outcomes has also been examined in individuals with arthritis [7, 8, 11–19]. Household income was found to be the most salient SES variable associated with arthritis-related physical health outcomes [7]. Studies have also found that in socially disadvantaged neighborhoods the prevalence rate of arthritis is higher [11]. However the impact of multiple SES indicators on mental health outcomes in those with arthritis remains understudied.

Those studies that have focused on mental health outcomes have mostly used a depressive symptoms scale as the only measure for mental health [18–21]. For example, a study examining both community and individual SES in systemic lupus erythematosus (SLE) related outcomes found that when using the Center for Epidemiological Studies Depression (CES-D) scale, rates of depressive symptoms were higher for individuals living in an impoverished area even after adjusting for individual level factors [19]. Using only depression as a proxy for mental health may be problematic, in that certain depressive symptoms (e.g., sleep disturbances, musculoskeletal complaints, back pain, and fatigue) can often be confounded with somatic symptoms known to accompany rheumatic conditions. Moreover, the CES-D is designed specifically to measure depressive symptomology [22]. Utilizing scales that only capture one mental health construct (i.e., depression) minimizes the opportunity to better understand the risk of poor mental health outcomes among a financially vulnerable population and potentially results in an underestimation of the public health impact. Therefore, further research examining the relationship between both individual and community level SES on a broader range of mental health outcomes in this context is warranted.

Arthritis-related studies to date have either focused on one measure of SES or one indicator of mental health. In light of the paucity of research on the association of individual and community level SES on multiple mental health outcomes, we contribute to the literature by building upon previous work to examine the associations between four individual level SES measures (educational attainment, household income, occupation, and homeownership) and a census-based community level SES measure (poverty rate) with three mental health outcomes in individuals with self-reported arthritis. Our objective is to further understand this relationship in effort to identify high risk populations, as well as to make intervention and policy recommendations that ultimately address pervasive health disparities. Notably, Healthy People 2020, a national public health initiative, has included reducing the proportion of individuals with arthritis reporting poor mental health or psychological distress as a key objective [23]. It is imperative, therefore, to understand the impact of contributing factors (e.g., individual and community level SES) that may place individuals with arthritis at an increased risk for poor mental health. For that reason, this study is timely and could serve as a vital piece to this burgeoning area of research.

## 2. Methods

### 2.1. Participants and Procedure

**Figure 1:**
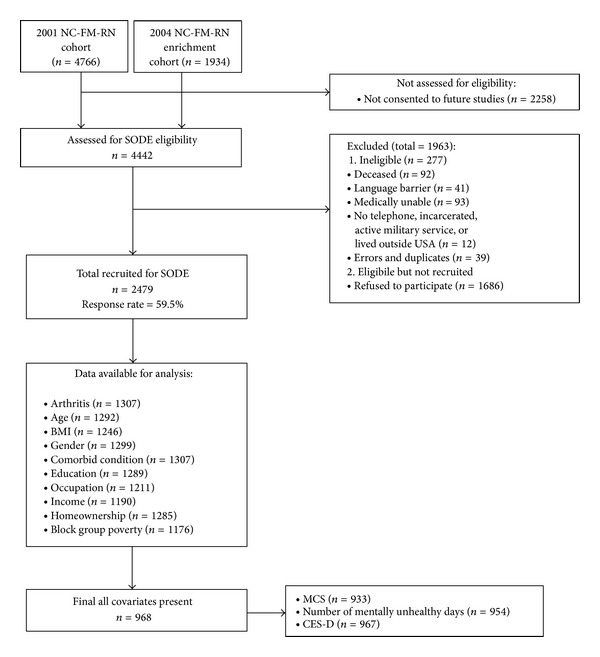
Participant recruitment and participation. NC-FM-RN: North Carolina Family Medicine Research Network; SODE: Individual and Community Social Determinants of Arthritis Outcomes Study; BMI: body mass index; MCS: mental component summary; CES-D: Center for Epidemiological Studies Depression.

Community-dwelling adults age 18 and above were recruited from the North Carolina Family Medicine Research Network (NC-FM-RN), a state-wide practice-based network [24]. The method used to extract participants from the original NC-FM-RN cohort for the purpose of this study is delineated (Figure 1). NC-FM-RN participants from 2001 and 2004-2005 who had agreed to be contacted for additional studies were invited to participate. Out of the 4,442 assessed for eligibility (i.e., had up-to-date contact information and spoke fluent English), approximately 60% of the population (*n* = 2,479) participated in a 45-minute telephone survey where they were asked to provide information on health status, health attitudes and beliefs, and demographics [24]. Participants provided written informed consent prior to enrollment, and the study was approved by the University of North Carolina at Chapel Hill Biomedical Institutional Review Board.

This paper focuses on 1,307 participants self-reporting a doctor-diagnosed case of arthritis indicated by using the single item question (Have you ever been told by a doctor or other health professional that you have some form of arthritis, rheumatoid arthritis, gout, lupus, or fibromyalgia?) from the Behavioral Risk Factor Surveillance System (BRFSS) 1996–2010 Questionnaire [25]. This single-item question, which includes the case definition of arthritis, has been used by both the BRFSS and National Health Interview Survey (NHIS) as a way to estimate national population prevalence rates of arthritis [26]. While the case definitions used by BRFSS and NHIS are less stringent than clinical case definitions, self-reports of arthritis have been shown to be valid [27].

### 2.2. Outcome Measures

#### 2.2.1. Overall Mental Health

Mental health was assessed using the MCS of the standard Medical Outcome Study's (MOS) Short Form survey (SF-12v2). Higher scores (range: 0–100) indicate better mental health. The MCS has been shown to be useful in screening for psychological distress and mental health disorders in general. The SF-12v2 and the SF-36 are highly correlated, and the SF-12v2 has been found to be reliable in general populations [28].

#### 2.2.2. Mental Health Related Quality of Life (HRQOL)

Mental HRQOL was assessed using the CDC HRQOL Healthy Days Measure [29]. Participants were asked, “Now thinking about your mental health, which includes stress, depression, and problems with emotions, for how many days during the past 30 days was your mental health not good?” Scores range from 0 to 30 with a higher score indicating worse health. Questions from the CDC have been recommended for research of this type and have good construct validity, acceptable criterion, and known group validity [30].

#### 2.2.3. Depressive Symptoms

The CES-D Scale, a valid measure for depressive symptoms [31], is a 20-item, self-report scale yielding scores ranging from 0 to 60, with high scores indicating high levels of depressive symptoms [22].

### 2.3. Predictor Variables

#### 2.3.1. Educational Attainment

Education was self-reported as the highest level of educational attainment on a seven-item scale. Responses were trichotomized as less than high school degree, high school degree or GED, and greater than high school (referent).

#### 2.3.2. Household Income

Participants reported their total annual family income with income categories ranging from less than $15,000 to more than $75,000. Responses were trichotomized as less than $15,000, between $15,000 and $45,000, and greater than $45,000 (referent).

#### 2.3.3. Occupation

Using an open-ended question, participants reported their current or last occupation. The descriptions were coded using the 2000 US Census occupation classification and placed into one of two categories: nonmanagerial/physically demanding (e.g., construction, farming, forestry or fishing, manual labor) or managerial/nonphysically demanding (e.g., sales or administration, management, technical, office; referent). Based on the census group descriptions and previous work that has included occupation as an SES indicator [7, 32, 33], managerial/nonphysically demanding in this context implies occupations similar to the aforementioned examples that oftentimes result in higher SES. Henceforth, the categories will be referred to as managerial or nonmanagerial.

#### 2.3.4. Homeownership

Participants were asked, “Do you own your own home?” with response options being either “Yes (referent) or No.”

#### 2.3.5. Community SES

Community level SES was determined by using MapMaker Plus 7.2 to match each participant's home address to their 2000 US Census block group; on average a block group consists of 1,000 residents [34, 35]. The percentage of the population living in a household with an annual income below the poverty level was used as an indicator of community poverty [36]. As suggested in some studies, block group characteristics have been shown to be better indicators of the immediate SES of the environment than census tract characteristics [37]. A poverty rate indicator was assigned low (referent), medium, or high based on tertiles (cut points: 7.5% and 14.1%). Residents in a specific census block group share the same community poverty rate.

### 2.4. Covariates

Previous research links age, race, gender, and health characteristics (i.e., body mass index [BMI] and comorbidities) to mental health outcomes. Age was measured as a continuous variable using participant self-reported date of birth. Participants were also asked to report their race/ethnicity (e.g., Black/African American, White). BMI (kg/m^2^) was a continuous measure that was calculated using self-reported height and weight. For this study, number of comorbid conditions is a sum of all self-reported nonarthritis conditions, excluding depression.

### 2.5. Statistical Analysis

Using STATA statistical software 11.0 [38] descriptive and multiple regression analyses were conducted to investigate the impact of both individual and community level SES on mental health outcomes in individuals with self-reported doctor-diagnosed arthritis (*N* = 1,307). Multiple imputation was used to replace missing values. Initially, regression analyses, not adjusting for site location, were conducted with the imputed values. The same analyses were conducted using a sample that only included cases with complete data on predictor variables (*N* = 968). The results were similar for both the sample with imputed values and the sample using case-wise deletion to address missing data. Moreover, except for gender, participants with missing data were not significantly different by predictor variables (i.e., demographics, SES) or outcome variables when compared to those with complete data. Therefore, for simplicity and to be consistent with previous research using these data, the analyses presented were conducted on 968 participants with complete data on SES predictor variables and covariates [7, 39] (Figure 1).

Predictor variables were introduced into multiple linear regression models in a hierarchical manner to determine the significance of each variable, both alone and relative to others. First, regression analyses were conducted to investigate the separate association of the four individual level and one community level SES predictor variables with each mental health outcome. Second, each individual SES measure was paired with the community SES measure in the model to determine if there were independent effects.

Finally, for the primary analyses, SES variables were sequentially added to the model in a stepwise manner based on a priori considerations [7]. We first entered occupation and homeownership (Block I), followed by community poverty (Block II), then educational attainment (Block III), and lastly household income (Block IV). Referent categories are as indicated previously. All models were adjusted for covariates. Analyses were then conducted to assess a minimally important difference for each statistically significant SES/mental association.

In these analyses, individuals are nested within their family practice, and therefore, we accounted for potential intraclass correlation created by the practice by generating Huber-White robust standard errors. This allowed for a more conservative approach over ordinary linear regressions when establishing the significance of parameter estimates [40, 41]. The relatively small sample size renders multilevel modeling numerically problematic (unstable). Census block groups were used solely to ascribe a community SES to the individual. Typically, a practice will draw patients from many block groups, but some residents from a single block group may attend different practices.

A large body of research has documented the moderating effects of race/ethnicity and SES on morbidity and mortality in the US [42]. We found no evidence of a race/SES interaction after testing for effect modification. Therefore, analyses were not stratified by race/ethnicity.

## 3. Results

The total sample (*N* = 968) was on average middle-aged, educated, non-Hispanic White females, with low household income, though the majority of participants reported homeownership status. Participants had an average BMI of 31 kg/m^2^ and approximately 2 comorbid conditions. On average they reported a MCS score of approximately 49, a CES-D score of approximately 12, and approximately 6 mentally unhealthy days per month (Table 1). Initially, correlation analyses between individual and community level SES and mental health status outcomes were examined. Pearson and Spearman correlations were small and ranged between 0.05 and 0.30, indicating that neither multicollinearity nor singularity was influential in the current analyses (correlation analyses are not presented). Preliminary regression analyses were conducted to examine the relationships between individual and community level variables alone and then with community level variables. Although preliminary results are discussed in this section, tables are only presented when all variables are considered in the hierarchical regression analyses (Tables 2–4).

### 3.1. Overall Mental Health

In effort to closely examine the impact of SES on overall mental health, we examined the independent effect of each variable on the MCS outcome measure. Educational attainment, household income, occupation, and community poverty all had statistically significant associations with MCS. Statistically significant changes in MCS scores ranged from 1.82 to 6.07 points. Those with less than high school educational attainment scored approximately 2.8 points less on MCS than those with greater than high school educational attainment. Individuals who reported a household income less than $15,000 in comparison to the referent group scored approximately 6 points lower on MCS. Participants with a nonmanagerial occupation reported approximately 3 points lower on the SF-12v2 MCS scale compared to their counterparts. Homeownership was not significantly associated with MCS scores. Similar to educational attainment, only one level of the community poverty rate indicator was significantly associated with MCS. Participants who live in a medium poverty area reported approximately 2 points less on the MCS to those living in low community poverty. The effect of each individual SES measure along with community level SES on mental health outcomes was examined in a separate series of regression models, adjusting for covariates. Findings were similar to results when each variable was entered independently. However, the effect of community poverty on change in MCS scores was no longer statistically significant. All statistically significant changes were based on *P* < .05.

To further understand the relationship of the SES variables with overall mental health, results from the hierarchical regression analyses are presented in Table 2. Having a nonmanagerial occupation resulted in scoring approximately 2.8 points less on MCS in comparison to those with a managerial occupation in Block I. When community poverty was added in Block II, educational attainment in Block III, and household income in Block IV, a significant negative association remained between occupation and MCS. In addition, there was an association between household income and MCS; the individuals with an annual household income of less than $15,000 scored approximately 5.2 points lower on the MCS.

The focus of our analyses was not to determine if individuals reached the empirically derived cut points for each measure, but to determine whether the statistically significant differences found in change scores for health status outcomes are of practical relevance. Therefore, we examined or calculated a minimally important difference (MID) in change scores for each scale. A change score of 2-3 points (i.e., 0.2-0.3 effect size) on the MCS scale constitutes a MID [43, 44] in the SF-36 which is highly comparable with the SF-12v2 [27, 45]. Based on these criteria we found that most of our findings specific to the MCS yielded minimally important differences resulting in a change of approximately 2 points or greater.

### 3.2. Mental Health Related Quality of Life (HRQOL)

Examining the impact of five indicators separately on mental health related quality of life, results indicated that those with less than high school educational attainment reported 2 additional mentally unhealthy days per month in comparison to those with greater than high school educational attainment. Those having less than $15,000 and/or a household income between $15,000 and $45,000 had significantly more mentally unhealthy days in comparison to the referent category. Moreover, participants who live in a medium poverty area reported approximately 1.6 additional mentally unhealthy days compared to those living in low community poverty. Next, the effect of each individual SES measure (separately) along with community level SES on mental health related quality of life was examined adjusting for covariates. Low educational attainment was marginally significant (*P* = .05) in indicating approximately 1.7 additional unhealthy days, and a medium level of community poverty was independently significant in indicating approximately 1.4 additional unhealthy days in comparison to those living in a more affluent community. With household income and community level poverty both in the model, having a household income of less than $15,000 resulted in 4 additional mentally unhealthy days, and medium level of community poverty accounted for 1 additional mentally unhealthy days in comparison to the referent group. All changes in scores were significant at the *P* < .05 level unless otherwise noted.

The impact of individual-level and community-level SES on mentally unhealthy days was then examined using hierarchical regression analyses (Table 3). There were no significant effects in Block I; however, medium community poverty was associated with a greater number of mentally unhealthy days in Block II. This was maintained in Block III, though no other independent effects were found. The significant independent effect of medium-level community poverty remained in Block IV, though slightly attenuated. Household income was also independently significant for mentally unhealthy days; participants with an annual household income of less than $15,000 reported approximately 4 additional mentally unhealthy days in comparison to those with a household income greater than $45,000.

Using the distribution-based method, research has indicated that a general MID for patient-reported health status outcome scales is between an effect size of 0.2 (small) and 0.5 (moderate) [46–48]. Therefore, based on the quasi-effect sizes calculated to determine MID in the CDC HRQOL change scores, mentally healthy days can be considered at or just below a MID in this study.

### 3.3. Depressive Symptoms

Models examining the effect of each SES measure on depressive symptoms were examined separately. Educational attainment, household income, occupation, homeownership, and community poverty all had statistically significant (*P* < .05) associations with depressive symptoms, with changes ranging from approximately 1.66 to 8.36 points. Next, the effect of each individual SES measure along with community level SES on depressive symptoms was examined in each model, adjusting for covariates. Results were similar, however; community poverty was no longer significant in any of the models paired with the single measure of individual level SES. Lastly, the effect of all five SES measures on depressive symptoms was examined (Table 4). In Block I, both a nonmanagerial occupation and not owning a home were independently associated with an increase in depressive symptoms, and effects remain, though slightly attenuated, in Blocks II and III. Moreover, those with less than a high school education reported greater depressive symptoms relative to those with a higher educational attainment in Block III. A significant independent association with depressive symptoms persists in Block IV with household income. Those with a household income of less than $15,000 report more depressive symptoms than those with greater than $45,000. The significant independent effects of all other SES variables are eliminated with the addition of income. To our knowledge, little-to-no research has set benchmarks for MIDs specific to the CES-D measures. Based on the calculations for MID, the changes in scores have not only a statistically significant impact but a change in scores that suggest practical relevance.

## 4. Discussion

### 4.1. Summary

To our knowledge, our study is the first to examine the impact of four measures of individual level SES and community level SES measures on multiple measures of mental health outcomes in individuals with arthritis as a way to further understand arthritis-related health disparities. Our findings support previous research that has consistently reported an association between low individual level SES and poorer arthritis-related health outcomes [9, 13, 19] but also reveal new information. Our results indicated that poorer mental health status in people with self-report arthritis of any type was associated with lower levels of SES. Specifically, having lower levels of educational attainment and income were associated with poorer mental health across all three measures; ultimately income was most strongly associated with poor mental health status. In addition, lower levels of SES (individual and community poverty) consistently had statistically significant associations with depressive symptoms. This finding is consistent with evidence from prior research that demonstrated an association between both individual (i.e., education and income only) and community level SES and mental health outcomes among individuals with SLE, a rheumatic condition [19]. Moreover, recent research using the clinic site to proxy individual level SES found that the clinic site location (i.e., public hospital versus tertiary clinic site) was associated with depressive symptoms in individuals with RA [18]. Our study shows that residing in a more impoverished community is associated with poorer outcomes in all three mental health measures.

Findings from this study build upon previous research, as similar findings have been observed despite the focus on single measures of SES or specific type of arthritis. For example, Harrison et al. [15] found that, when using the SF-36 Mental Component Summary Survey (MCS) to measure mental health outcomes in people with rheumatoid arthritis (RA) only, mental health scores worsened for those living in an area with increased social deprivation. Callahan et al. [13] examined the impact of individual and community SES on HRQOL and found poorer MCS scores in the low educational group and the highest poverty level group among those who self-reported arthritis. Community SES was also associated with mental HRQOL even after controlling for individual level factors. In addition to building upon earlier work, our findings provide a foundation to further understand the many underlying complexities associated with arthritis health-related health disparities.

### 4.2. Implications

While additional research is warranted to completely understand the complexities embedded in the SES and mental health outcomes relationship among individuals with arthritis, our study has both practice and policy implications. In an effort to provide optimal health care for individuals with arthritis, clinicians should take into account that low income, concomitant with the somatic symptoms associated with the condition, may exacerbate the compromise on one's mental health. For example, treatment recommendations that address both physical and mental health outcomes in individuals with low SES may mitigate existing disparities. Moreover, health care providers are encouraged to consider the unique impact of neighborhood level factors on the mental health of individuals with arthritis. Based on our findings that clearly indicate a link between depressive symptoms and all SES indicators, we theorize that individuals with lower SES and/or living in a community with limited resources may enter the health care system at a later progression of their disease and/or receive poorer quality health care based on resources available. Such factors may ultimately place this vulnerable group at a greater risk for depressive symptoms. Furthermore, residing in a more affluent community may be indicative of an individual's ability to afford needed goods and services, including health care, while residing in an impoverished community may limit access to optimal resources for good physical and mental health. Being able to identify an individual that may have an increased risk for poor mental health can result in healthcare providers engaging in best practices that would ultimately foster adequate healthcare and prevention leading to an overall reduction in the number of individuals with arthritis reporting poor mental health.

In addition, it is essential that policy makers identify ways to fund and implement programs and services that lead to health promotion and prevention. Understanding the combined and unique impact of individual and community level SES factors on mental health in individuals with arthritis can be essential in providing a rationale for allocating resources. Specifically, mental health programs should be developed that are available, accessible, and most importantly, affordable for a population that may be at a great risk for poor mental health. Increasing availability, access, and affordability can minimize disease burden and mitigate existing arthritis related health disparities.

Overall, our results suggest the importance of future research examining multiple measures of SES, as we observed that various combinations of individual and community level SES were independently associated with mental health outcomes. Greater understanding in measured/observed SES variation might enable researchers to design arthritis interventions targeting specific populations (e.g., those with low education, low income, or residing in impoverished communities). Public health practitioners, health care providers, and policy makers should consider targeted interventions as a way to improve health outcomes for those with arthritis at both the individual and population level.

### 4.3. Limitations

Our study has several limitations. Although our participants were asked to report on three types of arthritis, OA, RA, and Fibromyalgia, the majority of our sample (59%) reported being diagnosed with OA, and reduced power limited our ability to examine differences by arthritis-type. Future research may wish to examine this, as better understanding of how individual and community SES are associated with mental health by arthritis-type is especially important, given that previous research has found that RA patients have significantly more depressive symptoms than OA patients [49]. The cross-sectional nature of this paper precludes us from drawing conclusions on how accumulative lifetime effects of either individual level or community level poverty (either constant or variable) might influence mental health outcomes. Cross-sectional data also prevents the ability to determine directionality or assess reverse causality in the relationship of the variables of interest (i.e., SES and mental health). Therefore, future research should provide consideration to the dynamics of the SES/mental health relationship among those with arthritis. However, a primary strength is the inclusion of multiple independent SES measures in conjunction with community poverty, and the inclusion of multiple mental health outcome measures that have the ability to capture different mental health constructs. In addition, this study includes a large racially and geographically diverse sample.

## 5. Conclusion

In conclusion, our study findings suggest that SES measures, mainly individual income, play a strong role in arthritis-related mental health outcomes, particularly depressive symptoms. Though community poverty did not drive the associations, it remains an important factor influencing mental health. We must continue to examine SES at multiple levels in our quest to understand, mitigate, and eliminate health disparities among individuals with arthritis.

## Figures and Tables

**Table 1:** Participant characteristics.

Variable	*N* = 968	
	M	SD
Age (years)	56.86	13.67
Body mass index (kg/m^2^)	30.57	7.37
Comorbid condition count (#)	1.74	1.43
Gender (% female)	73.55	
Homeowner (%)	78.41	
Educational attainment (%)		
>HS	52.07	
HS	30.37	
<HS	17.56	
Nonmanagerial occupation (%)	49.28	
Household income (%)		
>$45,000	33.57	
$15,000–45,000	40.60	
<$15,000	25.83	
Community poverty rate	12.18	8.59
Community poverty (%)		
Low (<7.5%)	33.47	
Med (7.5–14.1%)	33.57	
High (>14.1%)	32.95	
Mental health status outcomes		
MCS (SF-12v2)	48.63	11.57
Mental unhealthy days	5.76	9.10
Depression (CES-D)	12.34	11.69

Note*:* sample size varies for outcomes: MCS, *n* = 933; mental unhealthy days, *n* = 954; CES-D, *n* = 967.

**Table 2:** Parameter estimates for all SES variables, *B* (95% confidence interval).

Variable	SF-12v2 MCS			
	Block I	Block II	Block III	Block IV
Nonmanagerial Occupation	−2.76∗∗ (−4.11, −1.41)	−2.64∗∗ (−4.06, −1.22)	−2.45∗∗ (−4.13, −0.77)	−1.78∗ (−3.36, −0.19)
Non-homeowner	−1.96 (−4.33, 0.41)	−1.84 (−4.20, 0.53)	−1.69 (−4.14, 0.77)	−0.52 (−3.19, 2.16)
Community poverty				
Medium (7.5–14.1%)		−1.28 (−3.03, 0.47)	−1.27 (−2.93, 0.39)	−1.14 (−2.79, 0.50)
High (>14.1%)		−0.80 (−3.03, 1.42)	−0.84 (−3.01, 1.33)	−0.28 (−2.37, 1.80)
Educational attainment				
HS			0.59 (−0.98, 2.17)	1.13 (−0.35, 2.61)
<HS			−1.04 (−3.18, 1.10)	0.40 (−1.72, 2.53)
Household income				
$15,000–45,000				−1.00 (−3.14, 1.14)
<$15,000				−5.23∗∗ (−8.46, −2.00)

Total *R* ^2^	0.12	0.12	0.12	0.14
*F*	29.96∗∗∗	30.17∗∗∗	34.91∗∗∗	36.12∗∗∗

Note*: * Each model is adjusted for age, gender, BMI, race, and comorbid count.

*P < .05; **P < .01; ***P < .001.

**Table 3:** Parameter estimates for all SES variables, *B* (95% confidence interval).

Variable	Mental unhealthy days			
	Block I	Block II	Block III	Block IV
Nonmanagerial Occupation	1.00 (−0.40, 2.40)	0.87 (−0.54, 2.28)	0.54 (−0.97, 2.04)	0.03 (−1.36, 1.42)
Non-homeowner	0.58 (−1.04, 2.20)	0.45 (−1.17, 2.07)	0.31 (−1.33, 1.95)	−0.58 (−2.16, 1.00)
Community poverty				
Medium (7.5–14.1%)		1.41∗ (0.26, 2.57)	1.34∗ (0.27, 2.41)	1.16∗ (0.12, 2.20)
High (>14.1%)		0.67 (−0.92, 2.27)	0.66 (−0.94, 2.27)	0.17 (−1.39, 1.73)
Educational attainment				
HS			0.05 (−1.36, 1.46)	−0.46 (−1.85, 0.92)
<HS			1.41 (−0.43, 3.26)	0.26 (−1.82, 2.33)
Household income				
$15,000–45,000				1.42 (−0.01, 2.85)
<$15,000				4.14∗∗ (1.79, 6.49)

Total *R* ^2^	0.12	0.12	0.13	0.14
*F*	16.56∗∗∗	15.04∗∗∗	12.24∗∗∗	15.19∗∗∗

Note: Each model is adjusted for age, gender, BMI, race, and comorbid count.

*P < .05; **P < .01; ***P < .001.

**Table 4:** Parameter estimates for all SES variables in blocks, *B* (95% confidence interval).

Variable	Depression CES-D			
	Block I	Block II	Block III	Block IV
Nonmanagerial Occupation	2.98∗∗ (1.32, 4.65)	2.86∗∗ (1.17, 4.56)	1.98∗ (0.25, 3.72)	1.16 (−0.49, 2.82)
Non-homeowner	2.82∗∗ (1.29, 4.34)	2.67∗∗ (1.14, 4.21)	2.44∗∗ (0.91, 3.97)	1.00 (−0.67, 2.67)
Community poverty				
Medium (7.5–14.1%)		1.05 (−0.57, 2.67)	0.78 (−0.7, 2.26)	0.51 (−0.94, 1.96)
High (>14.1%)		1.87 (−0.36, 4.10)	1.72 (−0.45, 3.90)	0.96 (−1.13, 3.04)
Educational attainment				
HS			1.61 (−0.00, 3.23)	0.79 (−0.96, 2.54)
<HS			3.19∗∗ (1.37, 5.00)	1.31 (−0.83, 3.44)
Household income				
$15,000–45,000				2.20 (−0.02, 4.42)
<$15,000				6.74∗∗ (4.43, 9.05)

Total *R* ^2^	0.15	0.16	0.16	0.19
*F*	33.40∗∗∗	34.91∗∗∗	33.78∗∗∗	57.93∗∗∗

Note: Each model is adjusted for age, gender, BMI, race, and comorbid count.

*P < .05; **P < .01; ***P < .001.

## References

[1] Centers for Disease Control and Prevention http://www.cdc.gov/chronicdisease/resources/publications/AAG/arthritis.htm.

[2] DeVellis BM (1995). The physiological impact of arthritis: prevalence of depression. * Arthritis and Rheumatism*.

[3] Keefe FJ, Abernethy AP, Campbell LC (2005). Psychological approaches to understanding and treating disease-related pain. *Annual Review of Psychology*.

[4] He Y, Zhang M, Lin EHB (2008). Mental disorders among persons with arthritis: results from the World Mental Health Surveys. *Psychological Medicine*.

[5] Hudson CG (2005). Socioeconomic status and mental illness: tests of the social causation and selection hypotheses. *American Journal of Orthopsychiatry*.

[6] Yu Y, Williams DR, Aneshensel CS, Phelan JC (1999). Socioeconomic status and mental health. *Handbook of the Sociology of Mental Health*.

[7] Callahan LF, Martin KR, Shreffler J (2011). Independent and combined influence of homeownership, occupation, education, income, and community poverty on physical health in persons with arthritis. *Arthritis Care and Research*.

[8] Harrison MJ, Farragher TM, Clarke AM, Manning SC, Bunn DK, Symmons DPM (2009). Association of functional outcome with both personal- and area-level socioeconomic inequalities in patients with inflammatory polyarthritis. *Arthritis Care and Research*.

[9] Robert SA (1998). Community-level socioeconomic status effects on adult health. *Journal of Health and Social Behavior*.

[10] World Health Organization (2003). *Social Determinants of Health: The Solid Facts*.

[11] Brennan SL, Turrell G (2012). Neighborhood disadvantage, individual-level socioeconomic position, and self-reported chronic arthritis: a cross-sectional multilevel study. *Arthritis care & Research*.

[12] Callahan LF, Shreffler J, Mielenz T (2008). Arthritis in the family practice setting: associations with education and community poverty. *Arthritis Care and Research*.

[13] Callahan LF, Shreffler J, Mielenz TJ (2009). Health-related quality of life in adults from 17 family practice clinics in North Carolina. *Preventing Chronic Disease*.

[14] Cañizares M, Power JD, Perruccio AV, Badley EM (2008). Association of regional racial/cultural context and socioeconomic status with arthritis in the population: a multilevel analysis. *Arthritis Care and Research*.

[15] Harrison MJ, Tricker KJ, Davies L (2005). The relationship between social deprivation, disease outcome measures, and response to treatment in patients with stable, long-standing rheumatoid arthritis. *Journal of Rheumatology*.

[16] Jacobi CE, Mol GD, Boshuizen HC, Rupp I, Dinant HJ, van den Bos GAM (2003). Impact of socioeconomic status on the course of rheumatoid arthritis and on related use of health care services. *Arthritis Care and Research*.

[17] Jolly M, Mikolaitis RA, Shakoor N, Fogg LF, Block JA (2010). Education, zip code-based annualized household income, and health outcomes in patients with systemic lupus erythematosus. *Journal of Rheumatology*.

[18] Margaretten M, Barton J, Julian L (2011). Socioeconomic determinants of disability and depression in patients with rheumatoid arthritis. *Arthritis Care and Research*.

[19] Trupin L, Tonner MC, Yazdany J (2008). The role of neighborhood and individual socioeconomic status in outcomes of systemic lupus erythematosus. *The Journal of Rheumatology*.

[20] Berkanovic E, Oster P, Wong WK (1996). The relationship between socioeconomic status and recently diagnosed rheumatoid arthritis. *Arthritis Care and Research*.

[21] Mair C, Diez Roux AV, Galea S (2008). Are neighbourhood characteristics associated with depressive symptoms? A review of evidence. *Journal of Epidemiology and Community Health*.

[22] Radloff LS (1977). The CES-D scale: a self-report depression scale for research in the general population. *Applied Psychological Measurement*.

[23] U.S. Department of Health Human Services Healthy people 2020. http://www.healthypeople.gov/2020/default.aspx.

[24] Sloane PD, Callahan L, Kahwati L, Mitchell CM (2006). Development of a practice-based patient cohort for primary care research. *Family Medicine*.

[25] Centers for Disease Control and Prevention Arthritis: data and statistics: BRFSS arthritis questions 1996–2010. http://www.cdc.gov/arthritis/data_statistics/brfss_questions.htm.

[26] http://www.cdc.gov/arthritis/data_statistics/faqs/case_definition.htm#5.

[27] Sacks JJ, Harrold LR, Helmick CG, Gurwitz JH, Emani S, Yood RA (2005). Self-reports have been shown to be valid for surveillance purposes: validation of a surveillance case definition for arthritis. *Journal of Rheumatology*.

[28] Ware JE, Kosinski M, Keller SD (1996). A 12-Item short-form health survey: construction of scales and preliminary tests of reliability and validity. *Medical Care*.

[29] Centers for Disease Control and Prevention Measuring healthy days: assessment of health-related quality of life. http://www.cdc.gov/hrqol/pdfs/mhd.pdf.

[30] Mielenz T, Jackson E, Currey S, DeVellis R, Callahan LF (2006). Psychometric properties of the Centers for Disease Control and Prevention Health-Related Quality of Life (CDC HRQOL) items in adults with arthritis. *Health and Quality of Life Outcomes*.

[31] Blalock SJ, DeVellis RF, Brown GK, Wallston KA (1989). Validity of the center for epidemiological studies depression scale in arthritis populations. *Arthritis & Rheumatism*.

[32] Cleveland RJ, Schwartz TA, Prizer LP (2013). Associations of educational attainment, occupation, and community poverty with Hip osteoarthritis. *Arthritis Care and Research*.

[33] Luong MN, Cleveland RJ, Nyrop KA, Callahan LF (2012). Social determinants and osteoarthritis outcomes. *Aging Health*.

[34] Krieger N (1991). Women and social class: a methodological study comparing individual, household, and census measures as predictors of black/white differences in reproductive history. *Journal of Epidemiology and Community Health*.

[35] Krieger N (1992). Overcoming the absence of socioeconomic data in medical records: validation and application of a census-based methodology. *American Journal of Public Health*.

[36] U.S. Census Bureau (2000). *Census 2000 Summary File 3: Census Population and Housing*.

[37] Roux AVD, Merkin SS, Arnett D (2001). Neighborhood of residence and incidence of coronary heart disease. *The New England Journal of Medicine*.

[38] StataCorp (2005). *Stata Statistical Software (Version 11.0)*.

[39] Baldassari AR, Cleveland RJ, Callahan LF (2013). Independent influences of current and childhood socioeconomic status on health outcomes in a North Carolina family practice sample of arthritis patients. *Arthritis Care and Research*.

[40] Hubbard AE, Ahern J, Fleischer NL (2010). To GEE or not to GEE: comparing population average and mixed models for estimating the associations between neighborhood risk factors and health. *Epidemiology*.

[41] Williams RL (2000). A note on robust variance estimation for cluster-correlated data. *Biometrics*.

[42] Anderson NB, Armstead CA (1995). Toward understanding the association of socioeconomic status and health: a new challenge for the biopsychosocial approach. *Psychosomatic Medicine*.

[43] Revicki DA, Menter A, Feldman S, Kimel M, Harnam N, Willian MK (2008). Adalimumab improves health-related quality of life in patients with moderate to severe plaque psoriasis compared with the United States general population norms: results from a randomized, controlled Phase III study. *Health and Quality of Life Outcomes*.

[44] Weiss SC, Kimball AB, Liewehr DJ, Blauvelt A, Turner ML, Emanuel EJ (2002). Quantifying the harmful effect of psoriasis on health-related quality of life. *Journal of the American Academy of Dermatology*.

[45] Ware JE, Kosinski M, Turner-Bowker DM, Gandek B (2002). *How to Score Version 2 of the SF-12 Health Survey (with a Supplement Documenting Version 1)*.

[46] Cohen J (1988). *Statistical Power Analysis for the Behavioral Sciences*.

[47] Norman GR, Sloan JA, Wyrwich KW (2003). Interpretation of changes in health-related quality of life the remarkable universality of half a standard deviation. *Medical Care*.

[48] Samsa G, Edelman D, Rothman ML, Williams GR, Lipscomb J, Matchar D (1999). Determining clinically important differences in health status measures: a general approach with illustration to the Health Utilities Index Mark II. *PharmacoEconomics*.

[49] Abdel-Nasser AM, Abd El-Azim SA, Taal E, El-Badawy SA, Rasker JJ, Valkenburg HA (1998). Depression and depressive symptoms in rheumatoid arthritis patients: an analysis of their occurrence and determinants. *British Journal of Rheumatology*.

